# Effect of Intracoronary Recombinant Human Prourokinase on Myocardial Perfusion and Clinical Outcomes in STEMI Patients Undergoing Primary PCI: A Randomized Controlled Trial

**DOI:** 10.31083/RCM39580

**Published:** 2026-08-14

**Authors:** Chonghuai Gu, Xin Hu, Haihong Liu, Rui Qiao, Xuejun Xiang, Haiyan Chen, Xin Zhao

**Affiliations:** ^1^Department of Cardiovascular, The Second Hospital of Dalian Medical University, 116023 Dalian, Liaoning, China; ^2^Department of Cardiovascular, Anqing Municipal Hospital, 246000 Anqing, Anhui, China

**Keywords:** myocardial perfusion, myocardial infarction, percutaneous coronary intervention

## Abstract

**Background:**

This study aimed to investigate whether intracoronary administration of recombinant human prourokinase (rhPro-UK) improves coronary blood flow, attenuates post-reperfusion inflammatory responses, and enhances clinical outcomes in patients with acute myocardial infarction.

**Methods:**

In this prospective, single-blind, randomized, controlled clinical trial, 136 ST-segment elevation myocardial infarction (STEMI) patients undergoing emergency percutaneous coronary intervention (PCI) at Anqing Municipal Hospital (August 2021–June 2023) were randomized 1:1:1 to intracoronary rhPro-UK (n = 44), tirofiban (n = 44), or saline groups (n = 48). Perioperative serum levels of creatine kinase-MB (CK-MB), high-sensitivity cardiac troponin T (hs-cTnT), interleukin-6 (IL-6), interleukin-8 (IL-8), tumor necrosis factor-alpha (TNF-α), and soluble suppression of tumorigenicity-2 (sST2) were measured. Serum sST2 was re-evaluated at 1 month (28 ± 7 days) post-PCI. Major adverse cardiovascular events (MACE) were assessed via clinic visits or telephone follow-up at 12 months.

**Results:**

The cohort had a mean age of 60.6 ± 11.5 years and was predominantly male (89.0%). At 12 months, the rhPro-UK group had the lowest MACE incidence (log-rank *p* = 0.039). Multivariable Cox regression confirmed reduced MACE risk in the rhPro-UK group (hazard ratio [HR] 0.22, 95% confidence interval [CI]: 0.05–0.99, *p* = 0.049). The final intra- and postoperative corrected TIMI frame count (cTFC) results demonstrated that both the tirofiban group and the rhPro-UK group exhibited superior efficacy in alleviating the slow/no-reflow phenomenon compared with the saline control group. At 48 hours post-PCI, rhPro-UK significantly reduced CK-MB and hs-cTnT levels compared to saline (*p* < 0.05). Both rhPro-UK and tirofiban groups exhibited lower IL-6, IL-8, and TNF-α levels at 24, 48, and 72 hours (*p* < 0.05). At 1-month follow-up, sST2 levels were markedly reduced in the rhPro-UK group (12.9 ± 3.3 pg/mL) versus tirofiban (18.7 ± 4.9 pg/mL) and saline (24.4 ± 15.7 pg/mL) (*p* = 0.001).

**Conclusion:**

Intracoronary rhPro-UK administration during emergency PCI in STEMI patients reduces post-reperfusion inflammation and fibrosis, limits infarct size, and improves clinical outcomes.

**Trial Registration:**

Chinese Clinical Trial Registry (ChiCTR; http://www.chictr.org.cn; Identifier: ChiCTR2100047095).

## 1. Introduction

Early reperfusion strategies—intravenous thrombolysis and primary percutaneous coronary intervention (pPCI)—significantly improve clinical outcomes. Despite its rapid accessibility, intravenous thrombolysis is limited by suboptimal reperfusion rates (50%–80%) and bleeding complications [1], establishing pPCI as the guideline-recommended optimal strategy [2]. However, pPCI is complicated by slow-flow/no-reflow phenomena in 20%–30% of cases [3], likely attributable to distal embolization of plaque debris and microvascular thrombosis during balloon/stent deployment. Current interventions focus on thrombus clearance to mitigate microvascular obstruction. STEMI-associated “red thrombi” (fibrin-rich with erythrocytes) may respond to fibrinolytic agents, while novel approaches combining pharmacological thrombolysis with mechanical revascularization aim to optimize reperfusion efficacy.

Sezer et al. [4] demonstrated that intracoronary low-dose streptokinase post-pPCI enhances drug delivery to thrombotic sites and improves microvascular perfusion. Subsequent studies confirmed that intracoronary alteplase or streptokinase administered immediately after pPCI enhances myocardial reperfusion and reduces infarct size [5]. Recombinant human prourokinase (rhPro-UK), structurally analogous to alteplase, exerts thrombolytic effects by converting to active urokinase at thrombus surfaces [6,7]. rhPro-UK is recommended for thrombolysis in STEMI, achieving high reperfusion rates with a low risk of intracranial hemorrhage [8]. Intracoronary administration of 10–20 mg rhPro-UK further enhances thrombus resolution and myocardial salvage without increasing bleeding complications [9,10,11,12,13].

Innovative drug delivery systems utilizing modified balloon catheters have emerged. Balaban et al. [14] employed perforated balloons to inject contrast during chronic total occlusion interventions, facilitating distal vessel visualization. Similarly, intracoronary nitroglycerin delivery via perforated balloons alleviates coronary spasm during PCI [15]. These devices enable localized, perpendicular drug infusion through micropores, optimizing endothelial penetration while minimizing systemic exposure. Tirofiban, widely used for slow-flow/no-reflow management, improves microvascular perfusion at high intracoronary concentrations [16,17,18,19].

To comprehensively evaluate the impact of reperfusion strategies, we selected biomarkers reflecting distinct pathophysiological pathways: creatine kinase-MB (CK-MB) and high-sensitivity cardiac troponin T (hs-cTnT) are established markers of myocardial injury, directly correlating with infarct size and prognosis; interleukin-6 (IL-6) and interleukin-8 (IL-8) are pro-inflammatory cytokines driving neutrophil infiltration and microvascular dysfunction during ischemia-reperfusion; tumor necrosis factor-alpha (TNF-α) amplifies oxidative stress and endothelial damage, exacerbating no-reflow phenomena; soluble suppression of tumorigenicity-2 (sST2), a decoy receptor for interleukin-33, is a prognostic biomarker linked to myocardial fibrosis and adverse ventricular remodeling. This study evaluates whether rhPro-UK delivered via a perforated balloon during pPCI reduces slow-flow/no-reflow incidence, limits infarct size, and improves coronary hemodynamics and clinical outcomes in STEMI patients.

## 2. Methods

### 2.1 Study Design

This prospective, single-blind, randomized, controlled clinical trial consecutively enrolled STEMI patients undergoing emergency pPCI at Anqing Municipal Hospital from August 2021–June 2023. Participants were randomized using the lottery method in a 1:1:1 ratio to three groups: the intracoronary recombinant human prourokinase group (n = 44), tirofiban group (n = 44), and saline control group (n = 48). The study protocol was approved by the Ethics Committee of Anqing Municipal Hospital, Anhui Province, and registered with the Chinese Clinical Trial Registry (PREPARE Trial, ChiCTR Registration Number: ChiCTR2100047095). Based on prior studies investigating intracoronary thrombolytics in STEMI patients, the primary endpoint was defined as the incidence of Major Adverse Cardiovascular Events (MACE) at 12 months post-PCI. Assuming a 25% MACE rate in the control group (saline) and expecting a 60% risk reduction with rhPro-UK intervention (to 10%), we set α = 0.05 (two-sided) and power = 90%. The final cohort included 136 patients (rhPro-UK: n = 44; tirofiban: n = 44; saline: n = 48), meeting the power requirement.

### 2.2 Inclusion and Exclusion Criteria

Inclusion criteria were: age 18–80 years; presence of myocardial ischemia symptoms with confirmed diagnosis of acute STEMI based on electrocardiographic findings, myocardial enzyme profiles, and diagnostic criteria outlined in STEMI guidelines established by the cardiology association; symptom onset ≤12 hours [20]; willingness to undergo reperfusion therapy; provision of written informed consent; and angiographically confirmed Thrombolysis in Myocardial Infarction (TIMI) flow grade ≤2 or TIMI thrombus burden grade ≥3 in the infarct-related artery.

Exclusion criteria included: receipt of rescue PCI following intravenous thrombolysis; history of intracranial hemorrhage; ischemic stroke within 3 months; suspected aortic dissection; active bleeding or bleeding predisposition; major head or facial trauma within 3 months; severe or uncontrolled hypertension; major trauma, surgery, or non-compressible vascular bleeding within 3 weeks; concurrent malignancy; severe hepatic or renal dysfunction; pregnancy or potential pregnancy; known allergy or contraindications to study medications or contrast agents; concurrent participation in other clinical trials; history of coronary artery bypass grafting (CABG); non-CABG patients with TIMI flow grade 3 and absence of angiographically confirmed thrombus (TIMI thrombus burden grade <3) in the infarct-related artery.

### 2.3 Patient Management

All enrolled STEMI patients received an aspirin loading dose (300 mg) and an ADP receptor antagonist loading dose (ticagrelor 180 mg or clopidogrel 300 mg) prior to reperfusion therapy, unless contraindicated. Coronary angiography was performed via transradial or transfemoral access using the standard Judkins technique, with multiple projections and angulations to fully visualize the culprit vessel. Lesions were classified as single-vessel or multi-vessel disease based on the number of coronary arteries with ≥50% stenosis. Pre-procedural TIMI flow grade and corrected TIMI frame count (cTFC) were recorded. A guidewire was advanced through the guiding catheter across the target lesion to the distal vessel. Predilatation was performed with a balloon catheter without contrast injection. The balloon was withdrawn outside the hemostatic valve (with the guidewire retained), and six symmetric perforations (three per side) were created using a syringe needle to fabricate a modified drug delivery system (perforated balloon). After flushing with saline and contrast evacuation, the perforated balloon was positioned with its proximal third at the lesion’s proximal edge and distal two-thirds within the lesion. Study agents—10 mL recombinant human prourokinase (10 mg in saline), 10 mL tirofiban (12.5 mg diluted to 50 μg/mL), or 10 mL saline—were infused over 5 minutes via the perforated balloon [20]. Post-infusion TIMI flow grade and cTFC (immediate post-PCI flow) were assessed. Immediate post-PCI flow was defined as coronary flow observed immediately after initial balloon inflation. Stent dimensions were operator-determined. Final TIMI flow grade and cTFC (terminal post-PCI flow) were documented. Successful pPCI was defined as residual stenosis <20% without in-hospital major adverse cardiovascular events (MACE). The study utilized recombinant human prourokinase (5 mg/vial, 500,000 IU; Shanghai Tianshi Pharmaceutical Co., Ltd.) and tirofiban (50 mL:12.5 mg; Lunan Pharmaceutical Group Co., Ltd.). Post-procedural therapy included dual antiplatelet therapy (aspirin plus tirofiban or clopidogrel). Adjunctive medications (statins, β-blockers, angiotensin receptor-neprilysin inhibitors, ACEI (angiotensin converting enzyme inhibitors), and/or ARB (angiotensin II receptor blockers)) were administered per clinical guidelines.

### 2.4 TIMI Flow Grade and cTFC Assessment

Coronary reperfusion was angiographically evaluated using standardized criteria: TIMI grade 0 indicates complete occlusion without antegrade flow distal to the obstruction; TIMI grade 1 reflects minimal antegrade flow with partial contrast passage through the occlusion and faint distal vessel opacification without complete vascular bed filling; TIMI grade 2 denotes partial reperfusion characterized by complete distal coronary opacification but delayed antegrade filling and clearance compared to non-infarct-related arteries; TIMI grade 3 represents complete reperfusion with rapid contrast filling and clearance. The cTFC was quantified by defining the first frame as contrast fully entering the vessel and the final frame as contrast reaching predefined distal landmarks: for the left anterior descending artery (LAD), the distal branch (or apical branches for elongated LAD); for the left circumflex artery (LCX), the distal bifurcation of the longest continuous segment; and for the right coronary artery (RCA), the first posterolateral branch originating from the posterior descending artery. Raw frame counts for LAD were divided by 1.7 to correct for its greater anatomical length compared to LCX and RCA. Slow flow/no-reflow phenomenon was angiographically defined as delayed distal vessel opacification meeting any one of the following criteria: (1) TIMI grade 2 flow (i.e., persistent contrast opacification requiring ≥3 cardiac cycles for vessel clearance); (2) corrected TIMI frame count (cTFC) >27 frames (with image acquisition standardized at 30 frames/second); (3) delayed opacification in ≥1 epicardial vessel.

### 2.5 Data Collection

Clinical records, procedural data, and follow-up documentation were systematically collected, including medical history (hypertension, diabetes, coronary artery disease, ischemic/hemorrhagic stroke, gastrointestinal disorders, chronic kidney disease, peripheral artery disease, malignancy, anemia), vital signs (heart rate, pulse, systolic/diastolic blood pressure), Killip classification, preoperative laboratory tests (complete blood count, hepatic/renal function, electrolytes, lipid profile, thyroid function, glucose, HbA1c, tumor markers), echocardiography (measured within 24 hours post-procedure), and PCI procedural data. Serum CK-MB and hs-cTnT levels were measured pre-PCI and at 4 h, 24 h, and 48 h post-PCI. IL-6, IL-8, and TNF-α levels were assessed at 24 h, 48 h, and 72 h postoperatively.

Serum sST2 was quantified using an immunofluorescence-based dual-antibody sandwich assay: samples were mixed with assay buffer, allowing fluorescent-labeled antibodies to bind ST2 antigen. The mixture was applied to test strips, diffused via capillary action, and captured by immobilized antibody pairs on the test (T) line, forming an ST2 antibody-antigen-fluorescent antibody complex. Fluorescence intensity, proportional to ST2 concentration, was analyzed by immunoassay analyzers. sST2 levels were measured at 4 h, 24 h, 48 h, and 72 h post-PCI.

### 2.6 Follow-Up

All patients underwent echocardiography and sST2 measurement at 1 month postoperatively (28 ± 7 days). At 12 months post-PCI, MACE including cardiac death, cardiac-related rehospitalization, angina, non-fatal myocardial infarction, and target vessel revascularization were assessed via outpatient visits or telephone follow-up, with the follow-up period concluding in August 2023. Cardiac death was defined as death resulting from severe cardiac dysfunction or failure caused by underlying cardiac disease or injury. Cardiac-related rehospitalization was defined as hospital admission triggered by decompensated chronic heart failure, acute coronary syndrome, arrhythmia, cerebrovascular accident, or peripheral vascular disease.

### 2.7 Endpoints

The primary endpoint of this study was the occurrence of MACE within 12 months after PCI. Secondary endpoints included the incidence of slow-flow/no-reflow phenomenon immediately after initial balloon dilation during PCI, as well as changes in serum levels of myocardial injury biomarkers (CK-MB and hs-cTnT), inflammatory biomarkers (IL-6, IL-8, and TNF-α), and myocardial fibrosis biomarkers (sST2) following the procedure.

The safety endpoint was the composite of any bleeding (Grades 1–5) as defined by the Bleeding Academic Research Consortium (BARC).

### 2.8 Statistical Analysis

Continuous variables were assessed for normality using the Shapiro-Wilk test, with normally distributed data expressed as mean ± standard deviation (x̄ ± s) and analyzed by ANOVA, while non-normally distributed data were reported as median (interquartile range) and analyzed using the Kruskal-Wallis H test. Categorical variables were presented as counts (%) and compared by χ^2^ test or Fisher’s exact test. Pairwise group comparisons were adjusted using the Bonferroni correction. This study utilized the Kaplan-Meier method to calculate the incidence of MACE within 12 months after PCI among the three groups, and intergroup comparisons were performed using the log-rank test. Multivariable Cox regression models were constructed, with the final adjusted model incorporating age, gender, smoking, alcohol use, hypertension, diabetes, stroke history, Killip class, heart rate, systolic blood pressure (SBP), diastolic blood pressure (DBP), hemoglobin, uric acid, glucose, triglycerides (TG), total cholesterol (TC), LDL-C, HDL-C, ALT, AST, and left ventricular ejection fraction (LVEF). Analyses were conducted using SPSS v24.0 (IBM Corp., Armonk, NY, USA), with two-sided *p* < 0.05 considered statistically significant.

## 3. Results

### 3.1 Clinical Characteristics

The study enrolled 136 patients, with 48 in the saline group, 44 in the tirofiban group, and 44 in the rhPro-UK group. The cohort had a mean age of 60.6 ± 11.5 years and was predominantly male (89.0%). Among the cohort, 50.7% presented with hypertension, 17.6% with diabetes mellitus, and 75.0% had a smoking history. Baseline clinical characteristics of the three groups are presented in Table 1. No significant differences were observed among the groups in age, gender, smoking, alcohol use, hypertension, diabetes, stroke history, Killip class, heart rate, SBP, DBP, hemoglobin, uric acid, or glucose levels (all *p* > 0.05). Additionally, the groups exhibited comparable lipid profiles (TG, TC, LDL-C, HDL-C), hepatic function (ALT, AST), and cardiac function (LVEF).

**Table 1.** **Baseline characteristics of the three patient groups**.

		Saline (n = 48)	Tirofiban (n = 44)	rhPro-UK (n = 44)	*p*-value
Age, years		60.8 ± 12.6	60.6 ± 11.8	60.5 ± 10.0	0.991
Male		42 (87.5)	37 (84.1)	42 (95.5)	0.217
Smoking		36 (75.0)	31 (70.5)	38 (86.4)	0.186
Alcohol		16 (33.3)	12 (27.3)	12 (27.3)	0.760
Hypertension		22 (45.8)	25 (56.8)	22 (50.0)	0.571
Diabetes		6 (12.5)	6 (13.6)	12 (27.3)	0.124
Stroke		4 (8.3)	3 (6.8)	2 (4.5)	0.764
Killip class					0.764
	I	44 (91.7)	41 (93.2)	42 (95.5)	
	II–IV	4 (8.3)	3 (6.8)	2 (4.5)	
HR, bpm		84.0 ± 24.8	82.9 ± 20.1	78.6 ± 15.6	0.418
SBP, mmHg		120.0 ± 19.6	123.8 ± 26.9	121.1 ± 29.8	0.764
DBP, mmHg		76.7 ± 14.1	80.6 ± 15.1	82.7 ± 18.2	0.179
Hemoglobin, g/L		146.3 ± 43.2	139.2 ± 16.1	150.6 ± 33.4	0.269
Uric acid, μmol/L		355.3 ± 90.6	334.7 ± 104.8	336.8 ± 92.3	0.525
Glucose, mmol/L		6.7 ± 2.4	6.4 ± 0.9	7.4 ± 3.1	0.142
TG, mmol/L		1.6 ± 1.8	1.3 ± 0.7	1.6 ± 1.0	0.427
TC, mmol/L		4.4 ± 1.2	4.5 ± 1.1	4.6 ± 2.3	0.826
LDL-C, mmol/L		2.5 ± 1.2	2.5 ± 0.9	2.5 ± 1.0	0.987
HDL-C, mmol/L		1.2 ± 0.4	1.2 ± 0.3	1.2 ± 0.3	0.832
ALT, IU/L		56.9 ± 30.8	52.3 ± 44.6	53.1 ± 34.5	0.813
AST, IU/L		284.6 ± 217.1	249.7 ± 213.8	288.5 ± 290.6	0.708
LVEF (%)					0.560
	<45	12 (25.0)	9 (20.5)	14 (31.8)	
	≥45	36 (75.0)	35 (79.5)	30 (68.2)	

HR, heart rate; SBP, systolic blood pressure; DBP, diastolic blood pressure; TG, triglyceride; TC, total cholesterol; LDL-C, low density lipoprotein cholesterol; HDL-C, high density lipoprotein cholesterol; ALT, Alanine transaminase; AST, Aspartate transaminase; LVEF, left ventricular ejection fraction.

Procedural characteristics are summarized in Table 2. All patients underwent transradial access. No significant intergroup differences were noted in contrast volume, intra-aortic balloon pump (IABP) use, pre-procedural TIMI flow grade, cTFC (all *p* > 0.05). But the final post-PCI TIMI flow and immediate and the final cTFC in rhPro-UK groups were significant better than other groups (all *p* < 0.05).

**Table 2.** **Peri-procedural clinical characteristics of the three patient groups**.

			Saline (n = 48)	Tirofiban (n = 44)	rhPro-UK (n = 44)	*p*-value
Contrast						0.034
	Isotonic		18 (37.5)	6 (13.6)	12 (27.3)	
	Hypotonic		30 (62.5)	38 (86.4)	32 (72.7)	
	Volume, mL		115.4 ± 38.1	120.9 ± 33.8	124.6 ± 42.0	0.513
IABP			4 (8.3)	4 (9.1)	4 (9.1)	0.989
TIMI						
	Pre-PCI					0.722
		0/1	42 (87.5)	38 (86.4)	36 (81.8)	
		2	6 (12.5)	6 (13.6)	8 (18.2)	
	Immediate post-PCI					0.055
		≤2	24 (50)	14 (31.8)	12 (27.3)	
		3	24 (50)	30 (68.2)	32 (72.7)	
	Final					0.001
		≤2	20 (41.7)	8 (18.2)	4 (9.1)	
		3	28 (58.3)	36 (81.8)	40 (90.9)	
cTFC (Frame)						
	Pre-PCI		55.8 ± 34.9	55.8 ± 27.1	54.4 ± 28.6	0.912
	Immediate post-PCI		46.1 ± 24.0	43.3 ± 20.2	31.7 ± 16.7	0.003
	Final		38.4 ± 18.6	33.8 ± 12.5	28.1 ± 11.4	0.004
Lesion vessel						0.742
	Single-vessel		16 (33.3)	17 (38.6)	18 (40.9)	
	Multi-vessels		32 (66.7)	27 (61.4)	26 (59.1)	
Stents						0.456
	1		36 (75.0)	32 (72.7)	28 (63.6)	
	≥2		12 (25.0)	12 (27.3)	16 (36.4)	

IABP, Intra-aortic balloon pump; TIMI, Thrombolysis in myocardial infarction; PCI, Percutaneous coronary intervention; cTFC, Corrected TIMI frame count.

The final intra- and postoperative corrected TIMI frame count (cTFC) results demonstrated that both the tirofiban group and the rhPro-UK group exhibited superior efficacy in alleviating the slow/no-reflow phenomenon compared with the saline control group.

### 3.2 Comparison of Biomarkers Among Groups

**Fig. 1.**
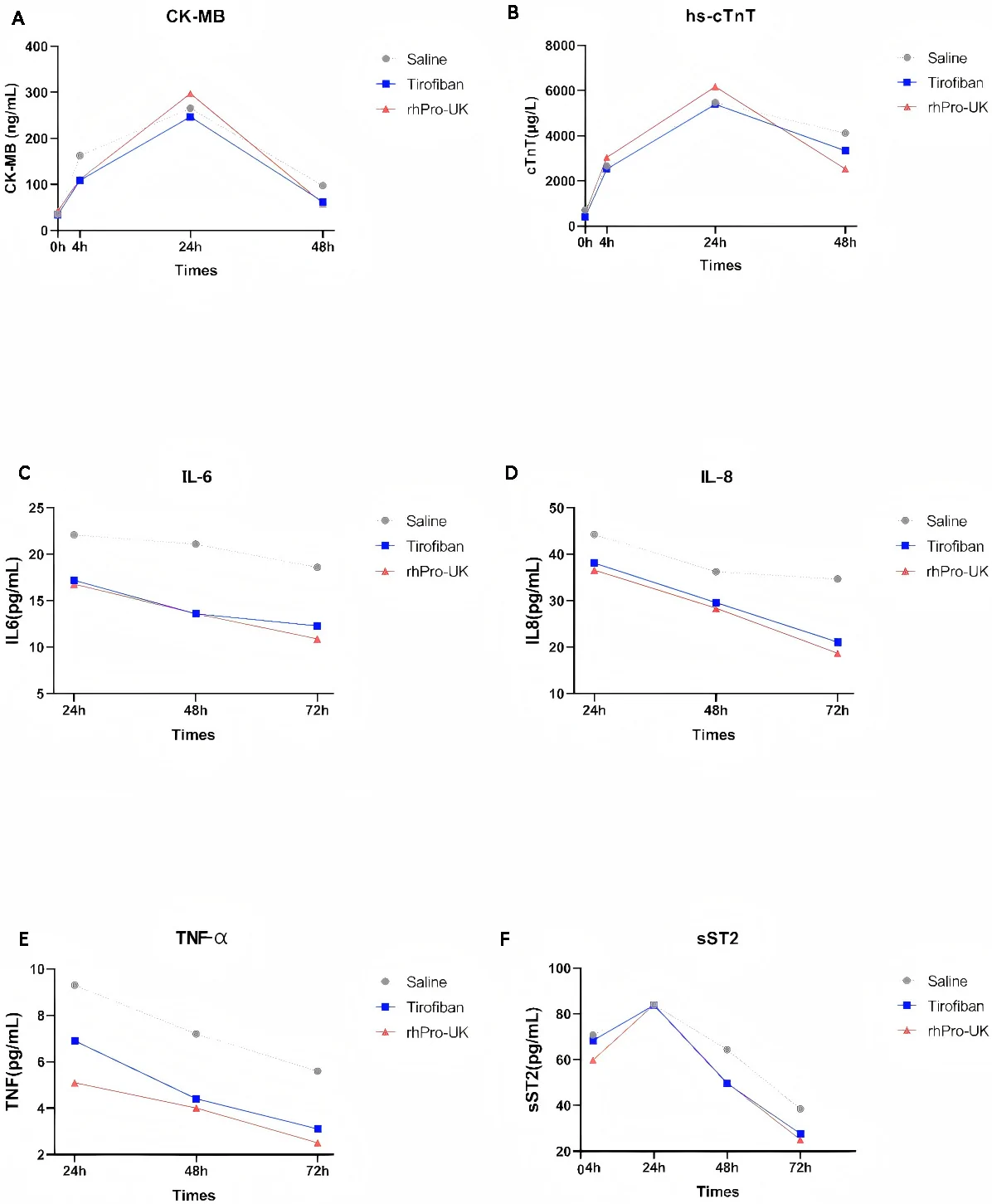
**The trend of serum biomarkers in the three groups**. (A,B) show the trends of CK-MB and hs-cTnT before PCI and at 4 h, 24 h, and 48 h after PCI. (C–E) show the trends of IL-6, IL-8 and TNF-α at 24 h, 48 h and 72 h after PCI. (F) shows the trend of sST2 at 4 h, 24 h, 48 h, and 72 h after PCI.

Serum biomarker levels across the three groups are summarized in Table 3 and Fig. 1. No significant differences in CK-MB levels were observed pre-PCI or at 4 hours post-PCI (*p* > 0.05). At 24 hours post-PCI, CK-MB values in the rhPro-UK and tirofiban groups decreased faster than in the saline group (297.4 ± 129.2 vs. 246.8 ± 145.4 vs. 265.6 ± 155.8 ng/mL, *p* = 0.465), though without statistical significance. By 48 hours post-PCI, the rhPro-UK group exhibited significantly lower CK-MB levels compared to the saline group (59.1 ± 22.1 vs. 97.6 ± 67.1 ng/mL, *p* = 0.005), while the tirofiban group showed no significant difference versus saline (74.1 ± 21.2 vs. 97.6 ± 67.1 ng/mL, *p* = 0.061).

**Table 3.** **Comparison of serum biomarkers between the three groups**.

	Times	Saline (n = 48)	Tirofiban (n = 44)	rhPro-UK (n = 44)	*p*-value
CK-MB (ng/mL)	Baseline level	35.6 ± 30.2	34.1 ± 20.3	42.8 ± 31.3	0.509
	4 hours after PCI	162.7 ± 105.6	109.1 ± 70.3	111.0 ± 57.3	0.077
	24 hours after PCI	265.6 ± 155.8	246.8 ± 145.4	297.4 ± 129.2	0.465
	48 hours after PCI	97.6 ± 67.1	74.1 ± 21.2	59.1 ± 22.1*	0.005
hs-cTnT (ng/L)	Baseline level	709.0 ± 868.0	400.2 ± 705.0	554.0 ± 814.0	0.673
	4 hours after PCI	2656.0 ± 1558.0	2527.0 ± 1424.0	3040.0 ± 1262.0	0.445
	24 hours after PCI	5467.0 ± 3723.0	5388.0 ± 3269.0	6172.0 ± 2165.0	0.668
	48 hours after PCI	4115.0 ± 1206.0	3214.0 ± 1180.0	2756.0 ± 1615.0*	0.030
IL-6 (pg/mL)	24 hours after PCI	22.1 ± 3.7	17.2 ± 4.4*	16.8 ± 5.5*	0.004
	48 hours after PCI	21.1 ± 7.0	13.6 ± 9.1*	13.6 ± 9.5*	0.033
	72 hours after PCI	18.6 ± 8.0	12.3 ± 3.6*	10.9 ± 4.0*	<0.001
IL-8 (pg/mL)	24 hours after PCI	44.3 ± 7.8	38.1 ± 9.3*	36.6 ± 7.0*	0.038
	48 hours after PCI	36.3 ± 3.3	29.6 ± 9.9*	28.4 ± 7.7*	0.0291
	72 hours after PCI	34.7 ± 14.2	21.1 ± 14.3*	18.7 ± 18.5*	0.016
TNF-α (pg/mL)	24 hours after PCI	9.3 ± 1.9	6.9 ± 3.3*	5.1 ± 2.5*	0.001
	48 hours after PCI	7.2 ± 3.0	4.4 ± 3.2*	4.0 ± 3.9*	0.026
	72 hours after PCI	5.6 ± 4.4	3.1 ± 2.8*	2.5 ± 2.8*	0.029
sST2 (pg/mL)	4 hours after PCI	70.8 ± 41.2	68.4 ± 41.3	59.8 ± 38.6	0.712
	24 hours after PCI	84.0 ± 59.4	83.8 ± 59.4	84.1 ± 19.0	0.703
	48 hours after PCI	64.4 ± 52.7	49.6 ± 45.4	49.9 ± 35.3	0.576
	72 hours after PCI	38.5 ± 15.7	29.7 ± 12.2	25.1 ± 11.8*	0.019

CK-MB, creatine kinase-MB isoenzyme; hs-cTnT, high-sensitivity cardiac troponin T; IL-6, Interleukin-6; IL-8, Interleukin-8; TNF-α, tumor necrosis factor-α; sST2, Soluble suppression of tumorigenicity-2. *Indicates *p* < 0.05 compared to the saline group.

For hs-cTnT levels, no intergroup differences were observed pre-PCI, at 4 hours, or 24 hours post-PCI (*p* > 0.05). At 48 hours post-PCI, both the rhPro-UK and tirofiban groups demonstrated significantly lower hs-cTnT levels compared to the saline group (2756.0 ± 1615.0 vs. 3214.0 ± 1180.0 vs. 4115.0 ± 1206.0 ng/L, *p* = 0.030), with the rhPro-UK group showing a significant reduction versus saline (*p* = 0.015), whereas tirofiban did not (*p* = 0.076). Both CK-MB and hs-cTnT levels exhibited an initial rise followed by a decline post-PCI (Fig. 1).

IL-6 levels in the rhPro-UK and tirofiban groups were significantly lower than in the saline group at 24, 48, and 72 hours post-PCI (*p* < 0.05), with no significant difference between rhPro-UK and tirofiban (*p* > 0.05). Serum IL-8 levels and trends mirrored those of IL-6 (Table 3 and Fig. 1). TNF-α levels in the rhPro-UK and tirofiban groups were significantly reduced compared to saline at 24, 48, and 72 hours post-PCI (*p* < 0.05), with no significant difference between the two active treatment groups (*p* > 0.05). TNF-α levels progressively declined post-PCI.

**Fig. 2.**
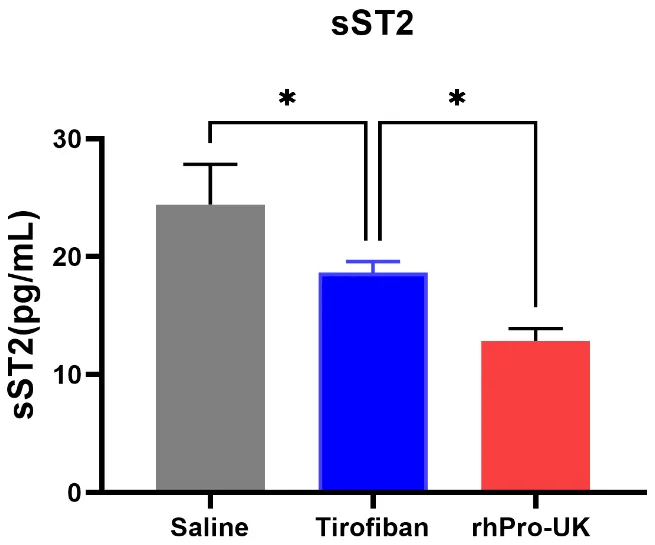
**Comparison of sST2 levels in the three groups at 1 month after PCI**. sST2, Soluble suppression of tumorigenicity-2. * indicates *p* < 0.05.

No significant differences in serum sST2 levels were observed among groups at 4, 24, or 48 hours post-PCI. At 72 hours post-PCI, both the rhPro-UK and tirofiban groups demonstrated lower sST2 levels compared to saline (25.1 ± 11.8 vs. 29.7 ± 12.2 vs. 38.5 ± 15.7 pg/mL, *p* = 0.019), with a significant reduction in the rhPro-UK group (*p* = 0.010) but not in the tirofiban group (*p* = 0.061). At 1-month follow-up (Fig. 2), the tirofiban group showed significantly lower sST2 levels than saline (18.7 ± 4.9 vs. 24.4 ± 15.7, *p* = 0.031 pg/mL), while the rhPro-UK group exhibited further reduction versus tirofiban (12.9 ± 3.3 vs. 18.7 ± 4.9 pg/mL, *p* = 0.024).

### 3.3 Major Adverse Cardiovascular Events at 1 Year Post-PCI

**Fig. 3.**
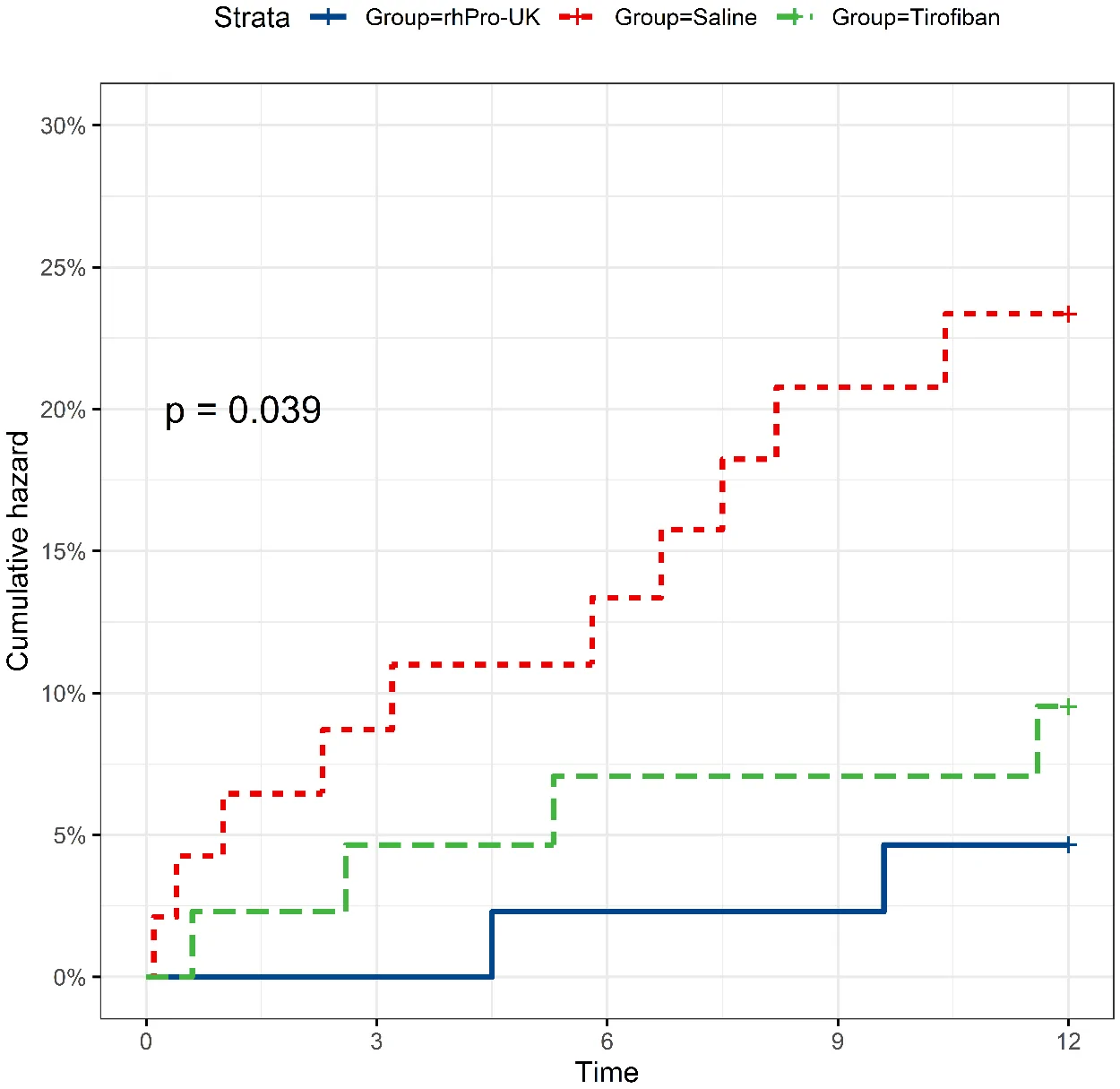
**Kaplan-Meier curve of MACE events at 1 year after PCI in the three groups of patients**. PCI, percutaneous coronary intervention; MACE, major adverse cardiovascular events.

All patients underwent meticulous follow-up, with MACE occurring in 10 (20.8%), 4 (9.09%), and 2 (4.55%) patients in the saline, tirofiban, and rhPro-UK groups, respectively. Kaplan-Meier curves for 1-year MACE are presented in Fig. 3. Both the rhPro-UK and tirofiban groups exhibited significantly lower MACE rates compared to the saline group (log-rank *p* = 0.039). Cox regression analysis (Table 4) demonstrated hazard ratios (HRs) of 0.41 (95% CI: 0.13–1.30, *p* = 0.129) for tirofiban and 0.20 (95% CI: 0.04–0.90, *p* = 0.036) for rhPro-UK versus saline. After adjusting for age, gender, smoking, alcohol use, hypertension, diabetes, stroke history, Killip class, heart rate, SBP, diastolic blood pressure (DBP), hemoglobin, uric acid, glucose, triglycerides (TG), total cholesterol (TC), LDL-C, HDL-C, ALT, AST, and left ventricular ejection fraction (LVEF), the rhPro-UK group maintained a significantly reduced MACE risk (adjusted HR 0.22, 95% CI: 0.05–0.99, *p* = 0.049) compared to saline.

**Table 4.** **Cox regression analysis of MACE events in the three groups one year after PCI**.

	Crude		Adjusted	
	HR (95% CI)	*p*-value	HR (95% CI)	*p*-value
Saline (n = 48)	Reference	Reference	Reference	Reference
Tirofiban (n = 44)	0.41 (0.13, 1.30)	0.129	0.44 (0.14, 1.43)	0.174
rhPro-UK (n = 44)	0.20 (0.04, 0.90)	0.036	0.22 (0.05, 0.99)	0.049

Adjusted model: adjusted for age, gender, smoking, alcohol, hypertension, diabetes, stroke, Killip class, heart rate, SBP, DBP, haemoglobin, uric acid, glucose, TG, TC, LDL-C, HDL-C, ALT, AST, LVEF.

Regarding safety endpoints, at the 1-year follow-up after PCI, BARC type 2–5 bleeding events occurred in 4 patients (8.3%) in the Saline group, 3 patients (6.8%) in the Tirofiban group, and 4 patients (9.1%) in the rhPro-UK group. No significant difference in bleeding risk was observed among the three groups (*p* = 0.924), as detailed in **Supplementary Fig. 1**.

## 4. Discussion

In this prospective randomized controlled trial, we investigated the clinical efficacy of intracoronary administration of low-dose rhPro-UK via a fenestrated balloon during pPCI in patients with STEMI. The principal findings were as follows: (1) Compared with the saline injection group, intracoronary administration of rhPro-UK via a fenestrated balloon significantly ameliorated the terminal slow/no-reflow phenomenon during the PCI procedure; (2) The rhPro-UK group demonstrated a more pronounced effect in reducing infarct size compared to the tirofiban group, while exhibiting comparable efficacy in attenuating inflammation and myocardial fibrosis; (3) At the 1-month follow-up, sST2 levels in the rhPro-UK group were markedly lower than those in both the tirofiban and saline groups; (4) During the 12-month follow-up, the rhPro-UK group exhibited a significantly lower incidence of MACE compared to the tirofiban and saline groups.

RhPro-UK is a guideline-recommended thrombolytic agent distinguished by its unique intrinsic enzymatic activity [21]. Its mechanism involves the conversion of plasminogen to plasmin specifically in the presence of thrombi within blood vessels, with plasmin further catalyzing the transformation of prourokinase to active urokinase. Previous studies comparing intravenous rhPro-UK thrombolysis with other thrombolytics (e.g., urokinase, alteplase) demonstrated superior efficacy of rhPro-UK in enhancing myocardial perfusion and safety profiles [22,23]. Current STEMI management guidelines explicitly highlight the advantages of rhPro-UK for thrombolytic therapy [24].

In this study, STEMI patients undergoing emergency pPCI received intracoronary thrombolytic administration via a fenestrated balloon prior to stent implantation. Post-PCI TIMI flow grade analysis revealed that compared to the saline control group, rhPro-UK delivery through the fenestrated balloon significantly ameliorated terminal slow/no-reflow phenomena during PCI. However, no significant difference was observed between the rhPro-UK and tirofiban groups, potentially due to the limited sample size of this study, which may have obscured detectable advantages in a small cohort. Immediate post-PCI cTFC comparisons demonstrated superior outcomes with rhPro-UK versus tirofiban, while final intraprocedural cTFC values indicated marked improvement in the rhPro-UK group over saline controls. Previous study has explored multiple intracoronary drug delivery routes, including guiding catheters, microcatheters, aspiration catheters, and infusion balloons [25]. However, agents administered via guiding catheters are often diluted in systemic circulation or diverted to non-target branches, rarely achieving adequate concentrations at coronary lesions. Microcatheter-based delivery necessitates guidewire removal and post-infusion repositioning, prolonging procedural duration and increasing complication risks. While novel infusion balloons enhance drug delivery efficacy, they elevate healthcare costs [9]. Additionally, both rhPro-UK and tirofiban groups exhibited more pronounced ST-segment resolution at 2 hours post-PCI, lower peak CK-MB levels, and fewer in-hospital TIMI major bleeding events versus controls [26]. Our findings similarly indicate that intracoronary rhPro-UK or tirofiban during PCI improves myocardial reperfusion in acute STEMI without increasing in-hospital bleeding rates, further validating the safety and reliability of intracoronary rhPro-UK. A key distinction of our study lies in the drug delivery device and the reduced rhPro-UK dosage. Current thrombolysis guidelines recommend an rhPro-UK loading dose of 20 mg intravenously, though systemic circulation may diminish coronary drug concentration [21]. Direct intracoronary injection theoretically enhances local plasma levels while minimizing systemic exposure. High-dose thrombolytics elevate bleeding risks; thus, we opted for a lower intracoronary dose of 10 mg rhPro-UK. Compared to administering 20 mg via aspiration catheters, fenestrated balloon delivery of 10 mg rhPro-UK may achieve a more favorable balance between mitigating slow/no-reflow and minimizing hemorrhagic complications. Nevertheless, our findings warrant validation through multicenter, large-scale studies akin to the ERUPTION trial.

Parameters assessing myocardial infarct size and prognosis (48-hour CK-MB and hs-cTnT levels) demonstrated that the rhPro-UK group achieved significantly greater reductions in infarct size compared to the tirofiban group. The rhPro-UK group also exhibited superior efficacy in mitigating inflammation and myocardial fibrosis (IL-6, IL-8, TNF-α) relative to the saline control. Furthermore, during the 1-year follow-up for MACE, the rhPro-UK group experienced fewer MACE occurrences, with rhPro-UK significantly outperforming tirofiban in reducing MACE incidence. A recent meta-analysis of intracoronary rhPro-UK administration, involving 1797 patients (897 receiving intracoronary rhPro-UK), confirmed the safety and efficacy of rhPro-UK combined with PCI in acute STEMI management [27]. Geng et al. [9] evaluated the therapeutic impact of intracoronary recombinant human prourokinase in STEMI patients via CK/CK-MB peak levels and MACE events, reporting reduced infarct size and improved prognosis. While their findings align with ours, notable methodological differences exist: their study utilized a single-aperture fenestrated balloon, whereas our approach employed a fenestrated balloon featuring two rows of six apertures, enabling faster and multidirectional drug delivery to lesion sites. Additionally, both studies administered drugs distal to the lesion, which inherently limits optimal plasma concentration at the target site. Although distal delivery enhances microvascular drug concentration compared to intravenous administration, it fails to achieve peak levels at the culprit lesion, potentially allowing residual microthrombi to migrate into distal microvessels and exacerbate microcirculatory dysfunction. Notably, some studies advocate direct thrombolytic injection into the thrombus core, enabling rapid dissolution of dense thrombotic burdens. Targeted intracoronary delivery not only lyses epicardial thrombi but also facilitates thrombolytic penetration into the coronary microcirculation, ensuring complete dissolution of microthrombi and debris, thereby achieving permeability-enhanced thrombus clearance from conduit to arteriolar vessels [28]. Proximal lesion administration of rhPro-UK offers dual advantages: direct thrombolysis at the lesion site and downstream microvascular distribution via blood flow, effectively addressing slow/no-reflow phenomena. The fibrin-specific enzymatic cascade of rhPro-UK enhances coronary flow restoration in thrombus-laden vessels, optimizing myocardial perfusion. These findings collectively indicate that intracoronary rhPro-UK in STEMI patients reduces slow/no-reflow incidence, improves microvascular function, limits infarct size, and enhances prognosis, consistent with our results [25,29].

sST2, a member of the interleukin-1 receptor family, exists in two primary isoforms: transmembrane (ST2L) and soluble (sST2). Interleukin-33 exerts cardioprotective anti-remodeling effects by inhibiting myocardial hypertrophy, fibrosis, and apoptosis. These effects are mediated via ST2L, where IL-33 binding activates a mechanically stimulated, cardioprotective fibroblast-cardiomyocyte paracrine system, facilitating adaptive responses during myocardial injury repair. Conversely, sST2 functions as a decoy receptor, antagonizing the IL-33/ST2L axis and blunting its cardioprotective properties [30]. Elevated sST2 levels correlate positively with cardiomyocyte hypertrophy and myocardial fibrosis, serving as a prognostic biomarker for adverse outcomes in chronic heart failure, including disease progression and mortality [31]. While prior studies predominantly focused on sST2’s role in heart failure prognosis, sST2 exhibits independence from BNP and remains largely unaffected by renal function. Combined assessment of sST2 and BNP enhances diagnostic accuracy and improves the evaluation of therapeutic efficacy and prognosis in heart failure [32,33,34]. Emerging evidence further links sST2 to infarct size, showing an inverse correlation with myocardial salvage index [35]. Patients with extensive myocardial infarction or microvascular obstruction demonstrate significantly higher sST2 levels [36], which are markedly elevated in the early phase of myocardial infarction [37]. Mechtoff et al. [38] reported a rapid decline in sST2 levels within 24 hours post-reperfusion therapy in STEMI patients, with elevated baseline sST2 associated with increased all-cause mortality risk during 12-month follow-up.

In this study, serial perioperative monitoring of sST2 levels revealed a declining trend post-PCI over time, which may be attributed to earlier sST2 peaking due to acute-phase intervention during myocardial infarction. Compared to the saline group, the rhPro-UK group demonstrated a significant reduction in sST2 levels at 72 hours post-PCI. At the 1-month follow-up, patients receiving intracoronary rhPro-UK via fenestrated balloon exhibited more pronounced sST2 reduction and a lower overall incidence of MACE than the tirofiban group, aligning with established correlations between sST2 levels and clinical prognosis. Park et al. [39] further reported that elevated sST2 levels during a 3-month follow-up in acute coronary syndrome patients significantly correlated with changes in left ventricular end-diastolic/end-systolic volume indices, though not with LVEF, identifying sST2 as a robust predictor of left ventricular remodeling.

Our study found that compared to intracoronary saline injection, rhPro-UK offers irreplaceable advantages when rapid dissolution of intracoronary thrombi is required to restore blood flow in patients with STEMI. Tirofiban achieves rapid and near-complete inhibition of platelet aggregation by blocking GP IIb/IIIa receptors. In contrast, rhPro-UK primarily targets the fibrinolytic system, contributing to coronary reperfusion.

### Limitations

As a single-center investigation with a small sample size and short follow-up duration, the findings may lack reliability due to the limited capacity to evaluate long-term prognostic outcomes. Although sST2 was incorporated to assess patient prognosis, the absence of more objective imaging modalities—such as cardiac magnetic resonance imaging (CMR)—to quantify myocardial infarct size restricts the robustness of the conclusions. Therefore, future studies with larger cohorts and comprehensive laboratory assessments are warranted to validate these findings.

## 5. Conclusion

In this study, we compared the effects of intracoronary administration of rhPro-UK, tirofiban, and saline in STEMI patients undergoing emergency PCI. By analyzing angiographic outcomes, serum biomarkers, and follow-up data, we demonstrated that rhPro-UK significantly reduced post-reperfusion inflammation and fibrosis levels, decreased myocardial infarct size, and improved clinical prognosis compared to the other groups.

## Data Availability

The data that support the findings of this study are available from the corresponding author, Xin Zhao, upon reasonable request. The data are not publicly available due to their containing information that could compromise the privacy of research participants.
