# Supplementary material for: Effect of Intracoronary Recombinant Human Prourokinase on Myocardial Perfusion and Clinical Outcomes in STEMI Patients Undergoing Primary PCI: A Randomized Controlled Trial
Source: Rev Cardiovasc Med. 2026 Aug 14;27(8):39580. doi: 10.31083/RCM39580 (PMC13540013; doi:10.31083/RCM39580)

Supplementary Figure 1. The percentage of patients with BARC bleeding events at 1 year after PCI in the three groups.

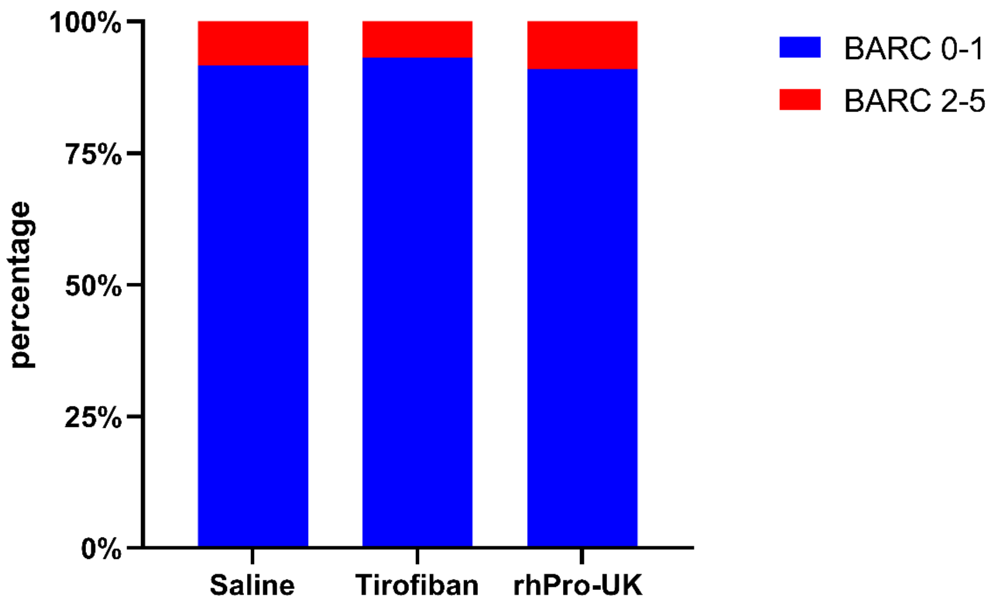

Supplement: Supplementary file 1 [file 2153-8174-27-8-39580-s1.zip › Supplementary Fig. 1.pdf]
